# A comprehensive protein interaction map and druggability investigation prioritized dengue virus NS1 protein as promising therapeutic candidate

**DOI:** 10.1371/journal.pone.0287905

**Published:** 2023-07-27

**Authors:** Qurrat ul Ain Farooq, Sara Aiman, Yasir Ali, Zeeshan Shaukat, Yasir Ali, Asifullah Khan, Abdus Samad, Abdul Wadood, Chunhua Li

**Affiliations:** 1 Faculty of Environmental and Life Sciences, Beijing University of Technology, Beijing, China; 2 National Center for Bioinformatics, Quaid-i-Azam University, Islamabad, Pakistan; 3 Faculty of Information Technology, Beijing University of Technology, Beijing, China; 4 School of Biomedical Sciences, The Chinese University of Hong Kong, Hong Kong, Hong Kong; 5 Department of Biochemistry, Abdul Wali Khan University, Mardan, Pakistan; The Islamia University of Bahawalpur Pakistan, PAKISTAN

## Abstract

Dengue Virus (DENV) is a serious threat to human life worldwide and is one of the most dangerous vector-borne diseases, causing thousands of deaths annually. We constructed a comprehensive PPI map of DENV with its host Homo sapiens and performed various bioinformatics analyses. We found 1195 interactions between 858 human and 10 DENV proteins. Pathway enrichment analysis was performed on the two sets of gene products, and the top 5 human proteins with the maximum number of interactions with dengue viral proteins revealed noticeable results. The non-structural protein NS1 in DENV had the maximum number of interactions with the host protein, followed by NS5 and NS3. Among the human proteins, HBA1 and UBE2I were associated with 7 viral proteins, and 3 human proteins (CSNK2A1, RRP12, and HSP90AB1) were found to interact with 6 viral proteins. Pharmacophore-based virtual screening of millions of compounds in the public databases was performed to identify potential DENV-NS1 inhibitors. The lead compounds were selected based on RMSD values, docking scores, and strong binding affinities. The top ten hit compounds were subjected to ADME profiling which identified compounds C2 (MolPort-044-180-163) and C6 (MolPort-001-742-737) as lead inhibitors against DENV-NS1. Molecular dynamics trajectory analysis and intermolecular interactions between NS1 and the ligands displayed the molecular stability of the complexes in the cellular environment. The *in-silico* approaches used in this study could pave the way for the development of potential specie-specific drugs and help in eliminating deadly viral infections. Therefore, experimental and clinical assays are required to validate the results of this study.

## 1.0. Introduction

Dengue is a vector-borne disease transmitted by mosquitoes, and it is currently the most serious arboviral infection, infecting approximately 400 million people worldwide annually, causing 25000 deaths every year [1–3]. In 2012, the World Health Organization (WHO) declared dengue as the most important mosquito-borne viral disease in the world, and despite the fact that it is of worldwide concern, 75% of the cases affected by dengue resided in the Asia-Pacific region [4, 5]. Dengue fever is endemic to Pakistan and is transmitted year-round with seasonal peaks. However, the number of recorded dengue cases is much higher in 2022 (between January and September) due to the worst floods in the country which started in mid-June. In Pakistan, 22,938 cases of dengue fever were recorded in 2017, more than 3,200 cases in 2018, 24,547 cases in 2019, and 3,442 cases in 2020, according to the National Institute of Health (NIH) in Islamabad. A total 48,906 cases, including 183 deaths, were recorded from January 1 to November 25, 2021. A rise in the number of cases was observed in 2021, especially in Lahore, Rawalpindi, and Islamabad. A total of 25,932 confirmed dengue cases and 62 fatalities have been reported nationally between January 1 and September 27, 2022. Seventy-four percent (74%) of these occurrences were recorded only in September. The distribution of cases by province was 83% (n = 21 777) of the total cases as of September 22, with 32% (n = 6888) of Sindh’s reported cases, 29% (n = 6255) of Punjab’s reported cases, 25% (n = 5506) of Khyber Pakhtunkhwa’s reported cases, and 14% (n = 3128) of Balochistan’s cases. Most of the cases in remote areas remained unrecorded and there might be the possibility of more cases as per local information. (https://www.who.int/emergencies/disease-outbreak-news/item/2022-DON414).

Dengue is a single-stranded positive-sense RNA virus belonging to the *Flaviviridae* family [6]. It has four serotypes, and currently, no therapy has been developed for dengue virus (DENV). In 2014, the Sanofi tetracelent vaccine (CYD-TDV) was developed against the dengue virus, but it has certain shortcomings, as it cannot be recommended for children less than 11 years of age [7, 8]. The genome of the dengue virus is approximately 11kb in size and is translated into 3 structural proteins (Capsid, M and E) and 7 non-structural proteins (NS1, NS2A, NS2B, NS3, NS4A, NS4B, and NS5) [6, 9, 10]. The study of interactomes is one of the utmost challenges in biomedicine, and is significant for the development of beneficial therapies for the treatment of human diseases [11–13]. Protein interaction networks can help us gain innumerable insights into the functional constituents of proteomes, life cycles of pathogens, and their involvement in disease pathways. Viruses infect humans through the interactions of their proteins with host proteins and it is necessary to gain detailed knowledge of virus-host interactions to understand the underlying infection mechanisms [14]. With the analysis of virus-host protein interactions, we can detect potential drug targets and host factors that could be enriched in a specific disease pathway and might intensify infections caused by the virus [15, 16]. Precise targeting of protein-protein interactions between viruses and their hosts is required for the development of antivirals. Because a viral pathogen hijacks its host cellular machinery and relies on the host for its replication, established intermolecular interactions between them are probably compulsory for its propagation [17]. In the past decade, there has been a lot of work done on PPI of several important viruses, and databases of viral-host protein-protein interactions have been filled with thousands of viral-human protein interactions. Previously, in our study on the protein interaction network of Hepatitis C virus (HCV) [18], Influenza A Virus (IAV) [19], and human papillomavirus (HPV) [20] with their host Homo sapiens, we identified some important proteins that can be used as potential drug targets for drug design aimed at diseases caused by HCV, IAV, and HPV infections.

The goal of this study is the identification and inhibition of potential therapeutic drug candidate proteins to halt and prevent the endemic DENV strains using system biology and cheminformatics approaches. We constructed an all-inclusive protein interaction network map of DENV with its host by integrating both small-scale and large-scale researches carried out experimentally till 2022. The idea of combining high-throughput protein interaction network data, followed by structural biology approaches for further investigations can lead to new approaches to identify highly therapeutic drug targets and development of novel drugs against emerging and remerging human pathogens. In this approach, every single study performed on the PPI of Dengue virus with human host till September 2022 is integrated. We also aimed to identify novel NS1 inhibitor by performing pharmacophore-based virtual screening of millions of small drug-like compounds available in public databases. *In-silico* ADME analysis was performed to study the pharmacokinetic properties of lead compounds. Furthermore, molecular docking studies were performed to validate the interactions and flexibility of NS1 with small drug-like molecules. Molecular dynamic simulations were performed to ensure stable molecular interactions and strong binding affinities of the receptor-drug molecules in cellular environment.

## 2.0. Material and methods

Consent statement is not applicable here as there are no participants involved in this study.

### 2.1. Protein-protein interaction (PPI) data collection

PubMed Advanced search featuring multiple keywords was used to gather all the data available on protein-protein interactions of DENV with its host, Homo sapiens. Out of the total 1254 results, we found our desired data in the 21 studies that exclusively contained PPI data of DENV-DENV and DENV-human. The data collected from these 21 studies were merged, we got 1195 unique interactions after removing duplicates. Table 1 lists the selected studies with their respective number of identified interactions.

**Table 1:** List of studies on PPI of DENV with human and DENV’s own proteins along with their respective numbers of interactions.

Research study	No. of Interactions identified in the study	References
Bhutkar *et al*., 2022	6	[21]
Ghildiyal *et al*., 2021	45	[22]
Dey *et al*., 2020	28	[23]
Silva *et al*., 2019	28	[24]
Srisutthisamphan *et al*., 2018	6	[25]
Giraldo *et al*., 2018	1	[26]
Hafirassou *et al*., 2017	220	[27]
Dey *et al*., 2017	535	[28]
Karyala *et al*., 2016	694	[29]
Dechtawewat *et al*., 2016	27	[30]
Diwaker *et al*., 2016	1	[31]
Cervantes *et al*., 2015	64	[32]
Zou *et al*., 2015	1	[33]
Zou *et al*., 2015	1	[34]
Pratap *et al*., 2013	1	[35]
Silva *et al*., 2013	50	[36]
Mairiang *et al*., 2013	53	[37]
Khadka *et al*., 2011	139	[38]
Doolittle *et al*., 2011	20	[39]
Umareddy *et al*., 2006	1	[40]
Chua *et al*., 2005	1	[41]

The interaction data collected in this study include only physical and direct interactions between DENV and human proteins, and have been verified by experimental methods, including Yeast 2 Hybrid (Y2H), Coimmunoprecipitation, mass spectrometry, GST pull-down assay, and co-transfection. The UniProt ID Mapping tool was employed to ensure that the network file loaded into Cytoscape has uniform data.

### 2.2. Analysis of the DENV-human PPI network

Network analysis was performed using several tools embedded within Cytoscape and other tools, including KEGG pathway and Gene Ontology (GO). The Cytoscape built-in tools used in the current research include network analyzer, CytoHubba [42], and Cytocluster [43]. To extract significant biological insights from the set of highly interacting gene products in the current study, we used the KOBAS software [44], which is a novel machine learning tool for functional enrichment analysis of gene sets. The analysis was performed with a corrected P-value <0.05.

In current study, we performed KEGG Pathway analysis of human proteins that were highly interacting with viral proteins to identify the pathways enriched with the selected group of genes. The Gene Ontology [45] analysis of the gene sets provides ontologies to describe non-overlapping attributes of the gene products, which are molecular function, biological process, and cellular components. GOnet [46] was used for gene ontology analysis of high-scoring human proteins. The enrichment P-values were computed using the Python goenrich package’s algorithm. Clustering analysis of the network was performed using the hierarchical clustering algorithm of Cytocluster.

### 2.3. Pharmacophore-based virtual screening

The Pharmit server was used for designing the pharmacophore models based on the 3D structure of DENV-NS1 protein (PDB ID: 4O6B). Pharmit server provides interactive screening of millions of compounds from built-in databases such as Molport, ZINC, ChEMBL, and PubChem [47]. The essential elements of an interaction are defined by the pharmacophore. The average of all atomic coordinates in their SMARTS chemical expression was calculated to obtain the coordinates of the pharmacophore model features. Pharmit performs Pharmer searches on the chosen databases. This search technique is similar to but distinct from geometric hashing and generalized Hough algorithms (two object recognition methods). In our study, 5 features were used to construct the model: two donors, two acceptors, and one hydrophobic. Out of millions of drug-like compounds, the screening results were limited to a meaningful level based on Pharmit scores and RMSD values to identify the best inhibitors [48].

### 2.4. Drug-likeliness and ADME assessment

Absorption, distribution, metabolism, and excretion (ADME) are the four key topics in pharmacokinetics. There is a relationship between physiological parameters and chemical structures; therefore, chemical descriptors can be used to calculate the pharmacokinetic properties. A good drug should completely and quickly absorb from the digestive tract, be clearly and unambiguously distributed to its target, metabolize in a way that does not immediately stop its activity, and finally gets out without causing any harm. The blood-brain barrier (BBB) was utilized to predict the penetration of blood into the brain following oral ingestion. The capacity of substances to penetrate this barrier was predicted by the skin permeability coefficient (log Kp). The lipophilicity of the drug was demonstrated by its logPo/w octanol-water partition coefficient. Low absorption and increased chemical metabolism are the effects of higher hydrophobicity. On the other hand, hydrophobic medications have a higher chance of attaching unfavorable hydrophobic macromolecules. Therefore, the hydrophobicity of the medication is crucial. Early ADME estimation during the discovery phase has been shown to significantly lower the percentage of pharmacokinetics-related failures during the clinical phases. SwissADME server [49] was used to evaluate the AMDET properties of the top hit compounds.

### 2.5. Molecular docking

Molecular docking analysis is essential for the prediction of the binding affinities and orientations of small molecules with the target protein [50]. The 3D-structures of the receptor and ligand molecules were prepared for molecular docking using the UCSF Chimera program [51], an interactive visualization and analysis program for molecular structures, and related data, such as density maps and sequence alignments. Autodock Vina version 1.2 [52] was used for molecular docking of the top hit compounds with NS1 (Table 1). AutoDock Vina is one of the most popular and swift open-source molecular docking tools. Simulating both straightforward and complicated docking operations is made easier with AutoDock Vina 1.2.0. The updated version offers python bindings, making it simpler to script for complex applications, such as virtual screening. The top hit compounds were subjected to molecular docking using AutoDcok Vina program, where the prediction of the binding site of the target protein and binding poses of query ligand were performed based on a novel curvature-based cavity detection approach (CurPocket) [53]. Discovery Studio Visualizer v.4.5 Client (Accelrys, San Diego, CA, USA) was used to carry out the analysis and visualization of the protein-drug molecular interactions. Molecular docking results were further validated by plotting the receiver operating characteristic (ROC) curve using area under the curve (AUC). The ROC curve was generated using the OrigenLab software (https://www.originlab.com/).

### 2.6. Molecular dynamic (MD) simulation

To study the conformational changes in protein dynamic motions, MD simulation was carried out using the Desmond program of the Schrödinger suite 2021–2 (Schrödinger, LLC, New York, NY, USA) with the Optimized Potentials for Liquid Simulation (OPLS4) force field [54] as implemented in previous studies [55–57]. The structures of DENV-NS1-C2 and DENV-NS1-C6 were solvated by creating a simulated triclinic periodic boundary box with extension of 10 Å in each direction and for each system, an explicit solvation model (Monte-Carlo equilibrated TIP3P: the transferable intermolecular potential 3 points) was used [58]. The mobility of all the covalent bonds and hydrogen bonds was regulated using the SHAKE algorithm [59] and Lennard-Johnes interactions with a cut-off of 10 [60]. To neutralize the entire system, additional counter ions (0.15 M of Na+Cl) were added during the solvation step. To obtain more realistic values for all the calculations, the ions for neutralization were placed 20 Å away from the bound ligand to the NS1 protein in the simulated system. The structures were subjected to energy minimization at a threshold of 25 kcal/mol/Å at 300 K temperature and 1 bar pressure achieved via the NPT ensemble class. Nosé–Hoover thermostat [61] and Martyna–Tobias–Klein barostat [62] methods were employed to maintain the conditions with a relaxation time of 12 ps during the simulation. The MD simulations were conducted for each system at a time interval of 50 ps. Long-range coulombic interactions were determined using the Particle Mesh Ewald (PME) method, while the covalent bonds with the hydrogen atoms were regulated using RRESPA motion integration package with a 2 fs inner time throughout the simulation [63]. A uniform density approach was used to determine long-range van der Waals interactions, and short-range electrostatic interactions were calculated using a cut-off of 9.0Å. The stability of each system was then evaluated based on the MD simulation trajectories by Schrödinger 2021–2 using the parameters Root Mean Square Deviation (RMSD), Root Mean Square Fluctuation (RMSF), Radius of gyration (Rg), H-bond occupancies, and Secondary Structure Elements (SSE).

### 2.7. Binding energies calculation

Prime MMGBSA (Molecular Mechanics Generalized Born Surface Area) was used to measure the binding free energy of Dengue NS1 protein in complex with the identified compounds [64, 65]. Using the Prime module of the Schrodinger suite and the OPLS 2005 force field, the binding free energies of receptor-ligand complexes were computed for 100 frames obtained after uniform time period for the whole simulation trajectory. The final energies were calculated based on the average energies from these 100 frames. The binding free energy of a ligand (L) to a protein (P) to form the complex (PL) is obtained as the difference.


$$\Delta G_{\left(binding\right)}=\Delta G_{\left(complex\right)}‐\Delta G_{\left(Protein\right)}‐\Delta G_{\left(Ligand\right)}$$

Where ΔG _*(complex)*_, ΔG _*(Protein)*_, and ΔG _*(Ligand)*_ stand for the free energies of a complex, a protein, and a ligand, respectively. ΔG _(*binding)*_ is the binding free energy.

## 3.0. Results

### 3.1. DENV-human protein-protein interaction network

**Fig 1:**
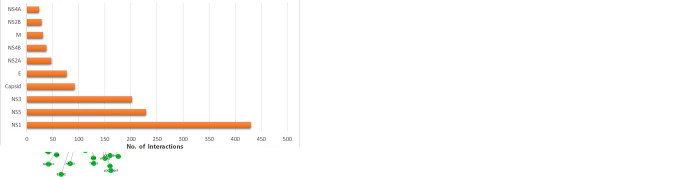
(a) Protein interaction network of DENV with human constructed using Cytoscape. The image shows the overall view of the network. There are 868 nodes in the network with 1195 interactions between them. Of the 868 proteins, 10 (purple) belongs to DENV while the remaining 858 are human proteins. Human proteins are shown in yellow, red, and orange. The size of the nodes represents the connectivity of the protein i.e., larger nodes are highly connected proteins in the network. The network is not very dense compared to any bacterial pathogen-host interaction network, and highly interacting nodes can be seen grouped into hubs. (b) Cluster of dengue viral protein NS3. Clusters in Cytoscape are created by a number of algorithms including k-medoid, hierarchical and k-means. (c) Bar chart representing the distribution of DENV proteins and their respective number of associations.

The PPI networks of DENV-DENV and DENV-human constructed in Cytoscape are shown in Fig 1A. The network was explored using network analyzer, that is capable of computing the average number of neighbors a node (protein) has in the network, the diameter of the network, and the high-scoring proteins in the network. It can also compute network density based on the total number of proteins and their interactions in the network. The statistical information for the current DENV protein interaction network is presented in Table 2.

**Table 2:** Important statistics of the DENV-human interaction network.

Attributes	Values
No. of Nodes	868
No. of Edges	1195
Avg. number of neighbors	2.74
High scoring interacting proteins in DENV	NS1, NS5, NS3
Network density	0.003
Network diameter	6
Clustering Coefficient	0.035
Shortest Paths	752556 (100%)

Using the CytoHubba plugin of Cytoscape, we obtained the node scores of the individual nodes (proteins) in the network. The non-structural protein NS1 of DENV has the maximum number (430) of interactions with human proteins, followed by the non-structural proteins NS5 and NS3 of DENV with 229 and 202 associations, respectively. Highly associated DENV proteins tend to form clusters in the network. Fig 1B shows the cluster formed by the interactions of dengue virus non-structural protein NS3 with host and other viral proteins. The minimum number of interactions a DENV protein has is 24 associations by NS4A. The specific number of associations for each DENV protein within the protein interaction network in the current study is shown in Fig 1C. S1 Table in the supplementary materials represents the viral proteins with highest percentages of interactions in the overall network. The full set of protein-protein interactions between DENV and Homo sapiens is shown in S1 Table.

**Fig 2:**
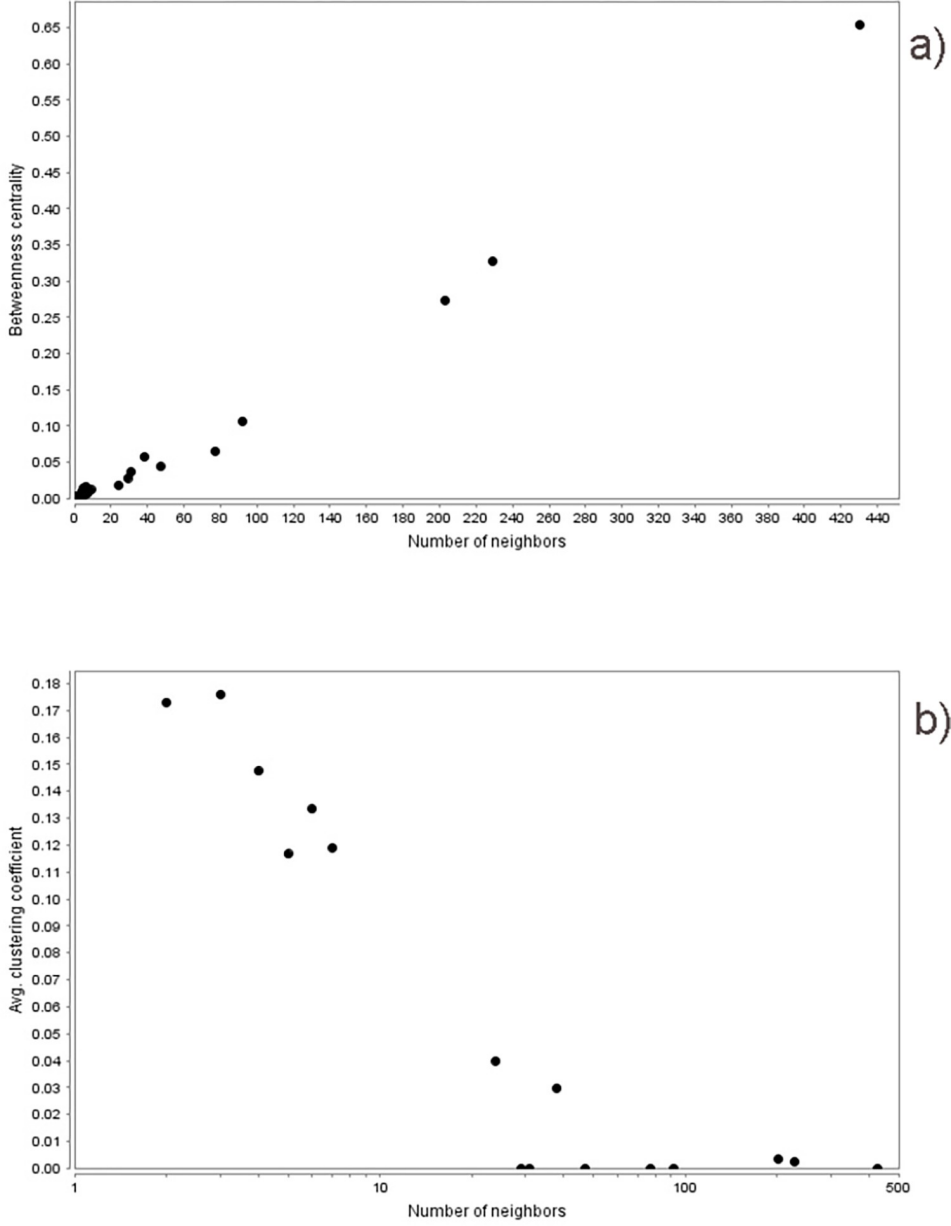
Graph showing a) betweenness centrality and b) clustering coefficient of the DENV-Human protein interaction network.

We identified various human proteins that interact with a greater number of dengue viral proteins. HBA1 and UBE2I interacted with 7 viral proteins, while CSNK2A1, RRP12, and HSP90AB1 interacted with 6 viral proteins. 12 human proteins were associated with 5 viral proteins in the network. S2 Table shows the list of the most highly interacting human proteins along with the viral proteins associated with them. The network was analyzed based on various topological parameters, including degree distribution, betweenness centrality, and clustering coefficient. Fig 2 represents the graph of betweenness centrality and clustering coefficient of the DENV-human protein interaction network with respect to its degree.

### 3.2. KEGG analysis of highly DENV-associated human gene set

**Fig 3:**
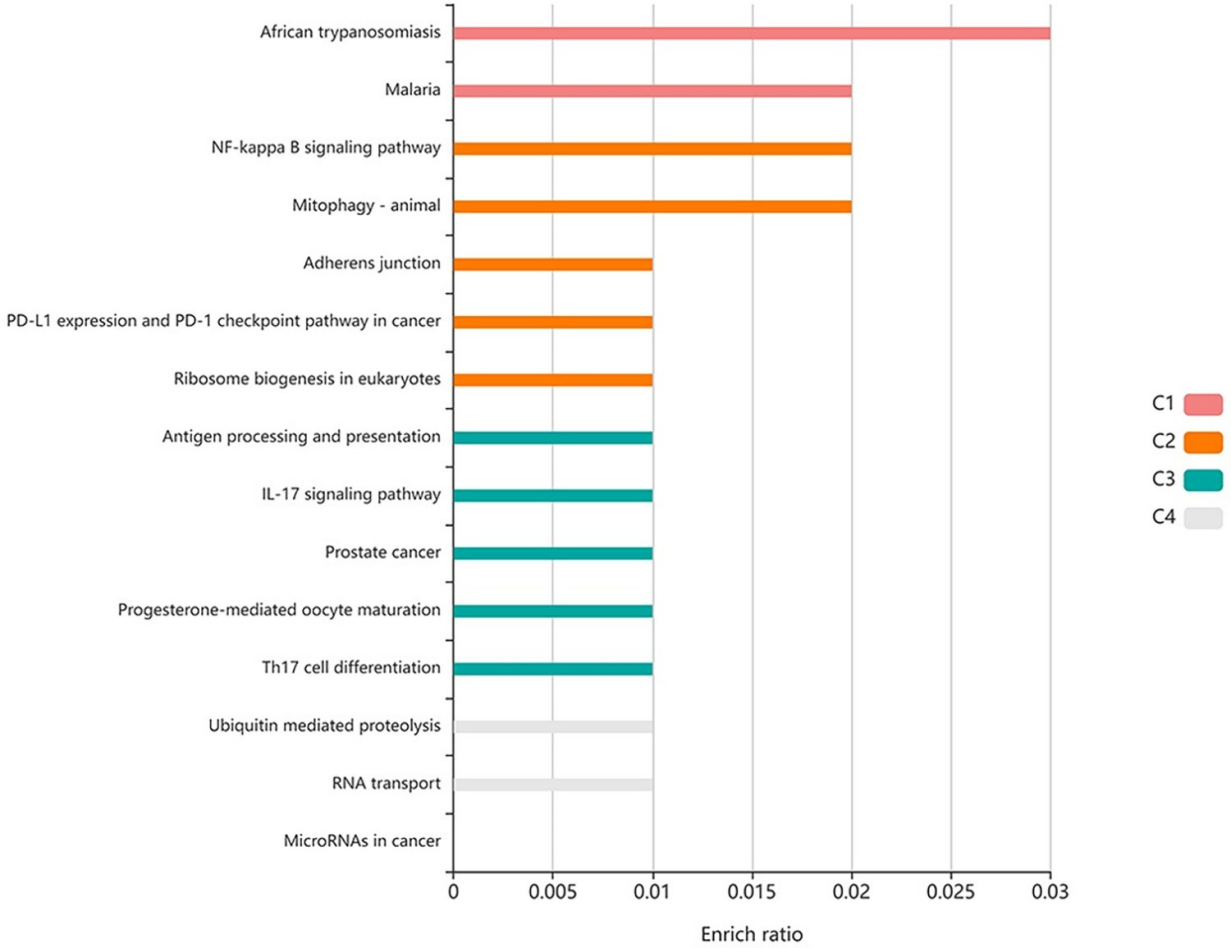
KEGG pathway analysis of the top 5 highly DENV-associated human proteins. The proteins are enriched in African trypanosmiasis (enrichment ratio: 0.03), which is an insect-borne disease that is transmitted by a fly. The results showed that proteins were also enriched in immune system NF-kappa B signaling pathway. Other pathways in which the gene products were enriched included malaria, mitophagy, and adherens junction. The bar indicates the enrichment ratio of proteins in a specific pathway.

**Fig 4:**
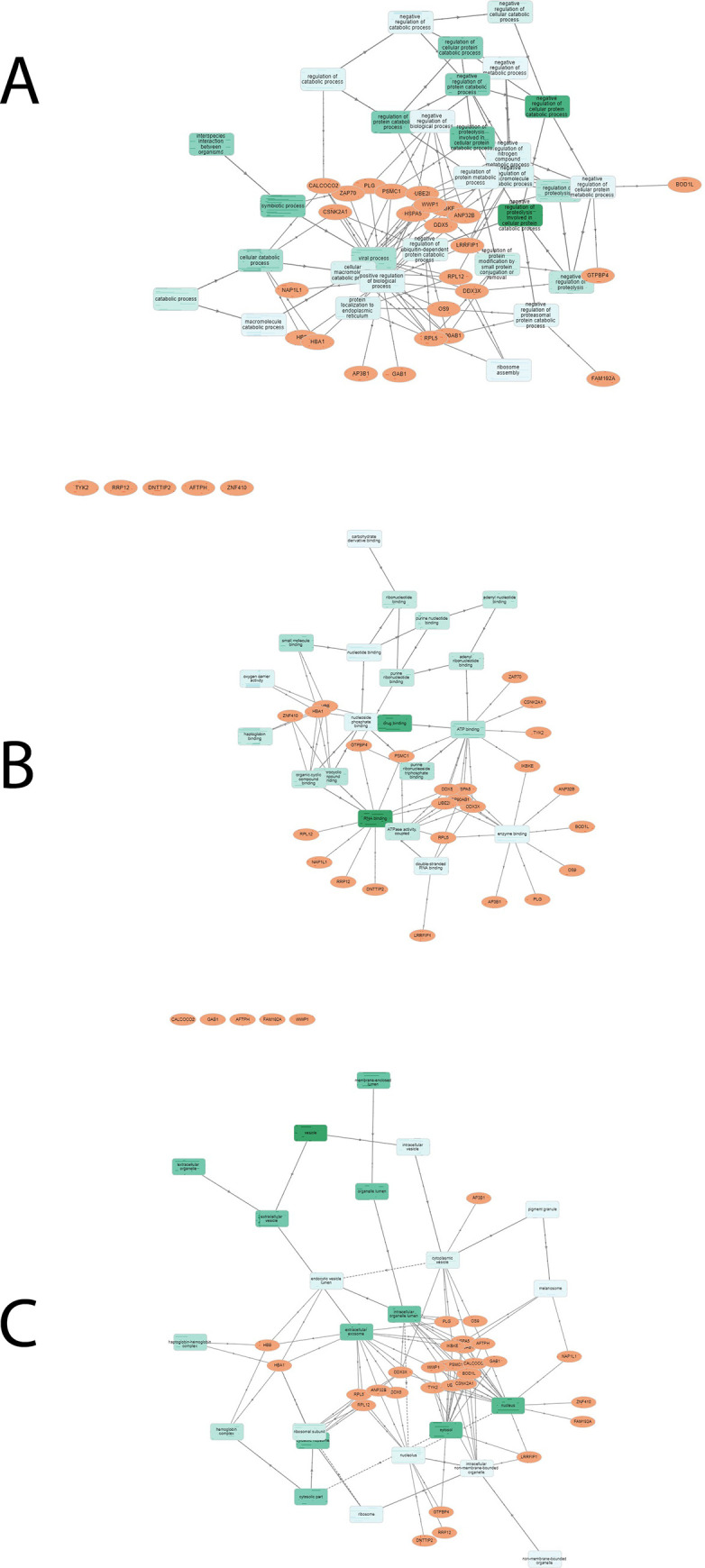
From top to bottom. The biological processes in which the genes are taking part include the negative regulation of cellular protein catabolic process (GO:1903363) and the regulation of proteolysis involved in cellular protein catabolic process (GO:1903050). These genes are involved in symbiotic process, regulation of cellular protein catabolic process, interspecies interactions between organisms, viral process, regulation of proteolysis, and several other important biological processes. The central interactive graph shows the molecular functions of the genes. These genes play important role in RNA binding (GO:0003723), drug binding (GO:0008144), and small molecule binding (GO:0036094). Other molecular functions of the genes in the gene set include ATP binding, purine ribonucleoside triphosphate binding, heterocyclic compound binding, and organic cyclic compound binding. The third graph shows that the genes were located in the vesicle, nucleus, cytosol, intracellular organelle lumen, membrane-enclosed lumen, extracellular exosome, and other cellular components.

The human gene set with the maximum number of associations with viral proteins (S2 Table) was analyzed. KEGG analysis of the top 5 human gene products with the highest number of associations with viral proteins showed that the proteins were highly enriched in various other pathways, including the disease pathway, on the top of which is African trypanosmiasis. Other disease pathways in which the gene set was enriched included malaria and prostate cancer. These proteins were also enriched in the NF-κB pathway, which serves as a crucial mediator of inflammatory responses. Fig 3 shows the KEGG pathway analysis of the top 5 human proteins with the highest number of associations with viral proteins. KEGG analysis of the top 30 most highly interacting host proteins (S1 Fig) showed that they were actively involved in the transport of proteins from the cytoplasm to the exterior of the cell. We performed gene ontology analysis of the top 30 most highly DENV-associated human genes (Fig 4) and identified the biological processes in which the genes are involved, the cellular component of which they are a part, and their molecular functions.

### 3.3. Clustering analysis

**Fig 5:**
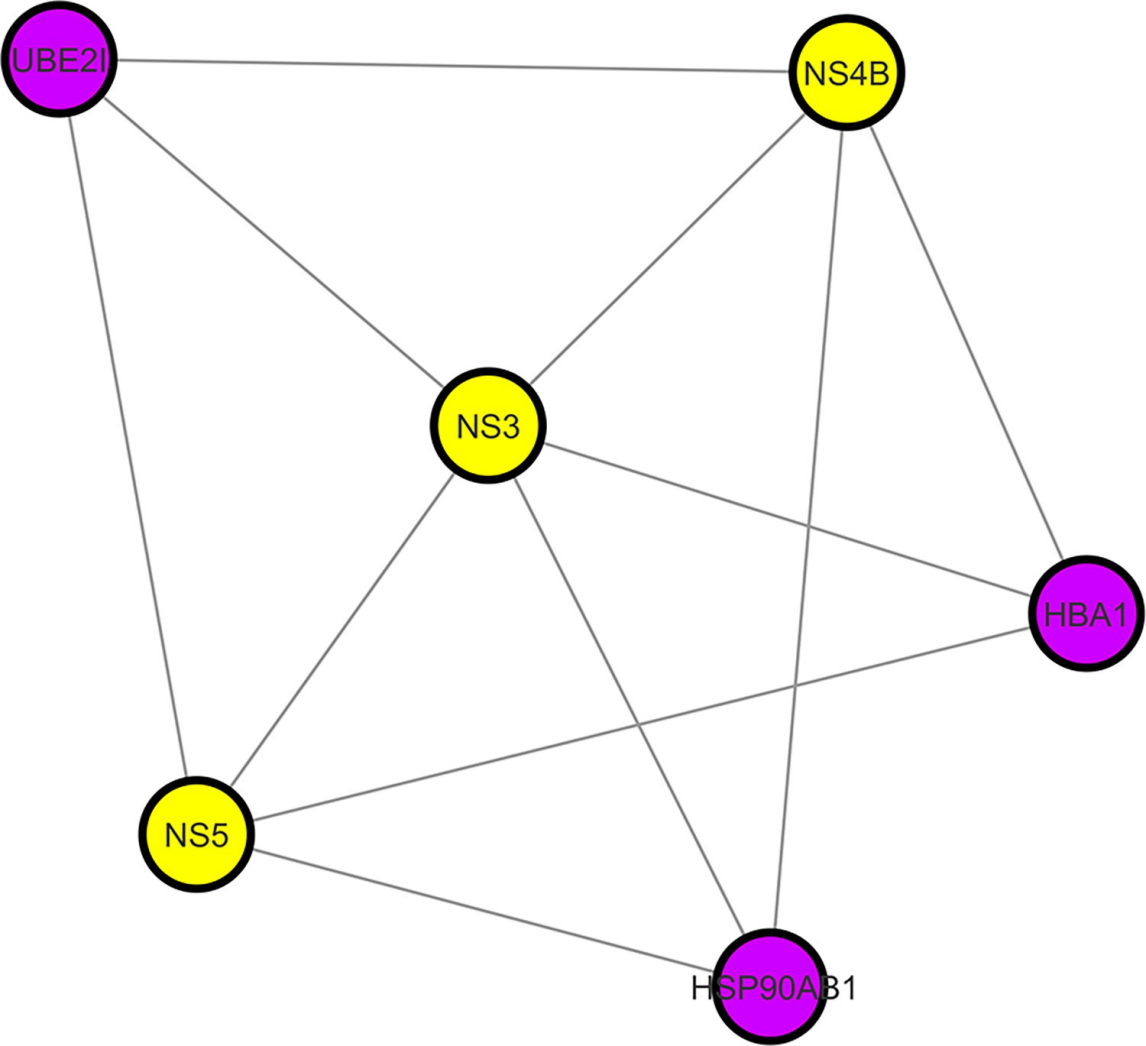
A cluster formed by CytoCluster using a hierarchical clustering algorithm. Viral proteins are represented by yellow nodes, whereas purple nodes represents human proteins.

Clustering analysis of the overall network was performed to find out functional protein complexes and modules. Cytoscape has various clustering algorithms based on different methods, including density-based, partition-based clustering, spectral-based, and hierarchical-based clustering methods. MCODE [66], CytoCluster [43], ClusterOne [67], and ClusterMaker [68] are the most commonly used Cytoscape apps for analyzing biological networks and for the identification of significant protein complexes and modules. Fig 5 shows a cluster constructed using CytoCluster in the protein interaction of DENV-human. The sub-network comprises of 6 nodes with viral proteins NS3, NS4B, and NS5, whereas UBE2I, HSP90AB1, and HBA1 belong to humans. We can obtain multiple clusters from a single protein interaction network using different clustering algorithms in Cytoscape based on different methods.

### 3.4. Pharmacophore-based virtual screening

**Fig 6:**
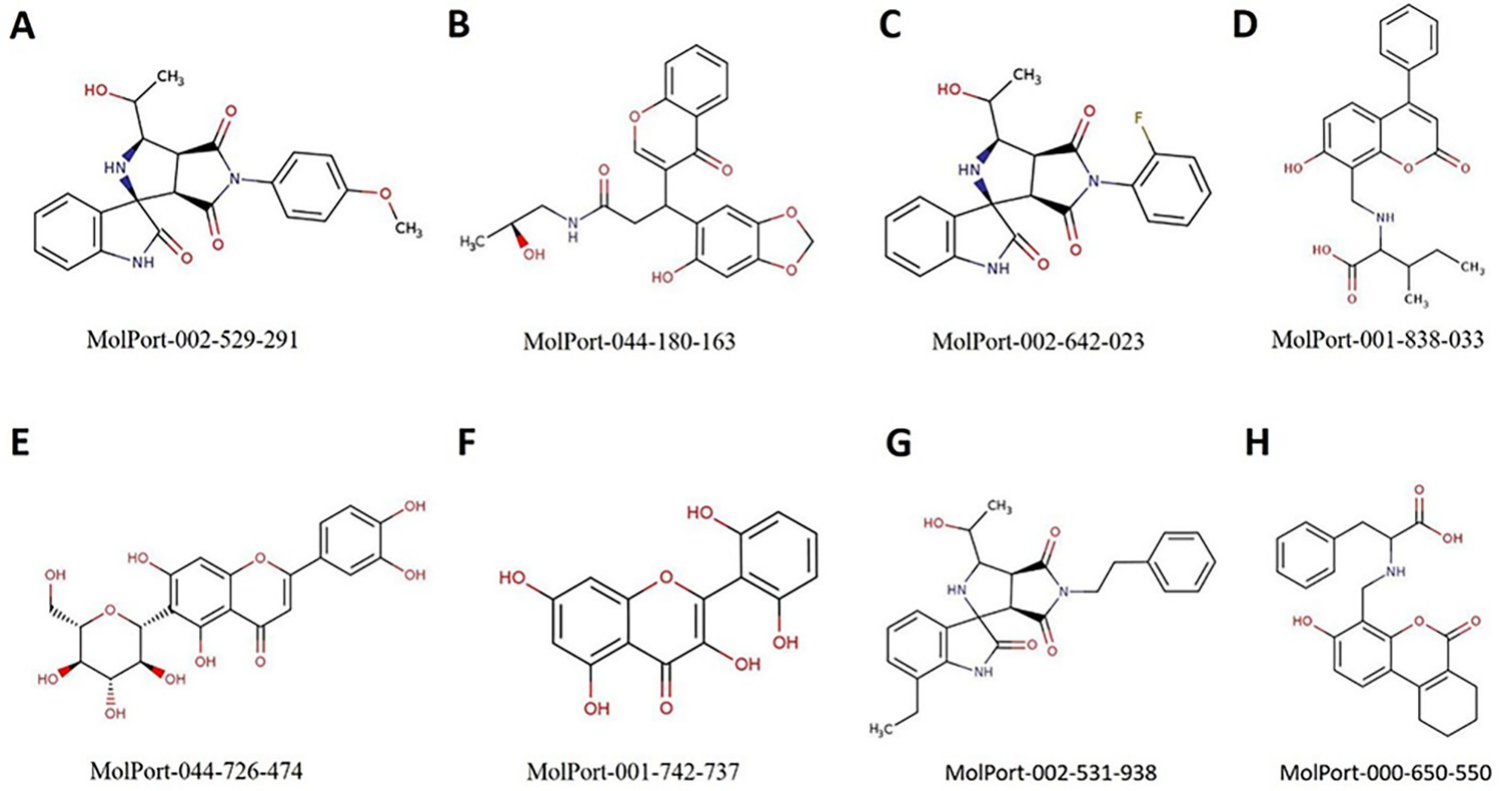
Chemical structures of the top hit compounds docked best with the binding pocket of NS1 based on Pharmit scores and RMSD values.

**Fig 7:**
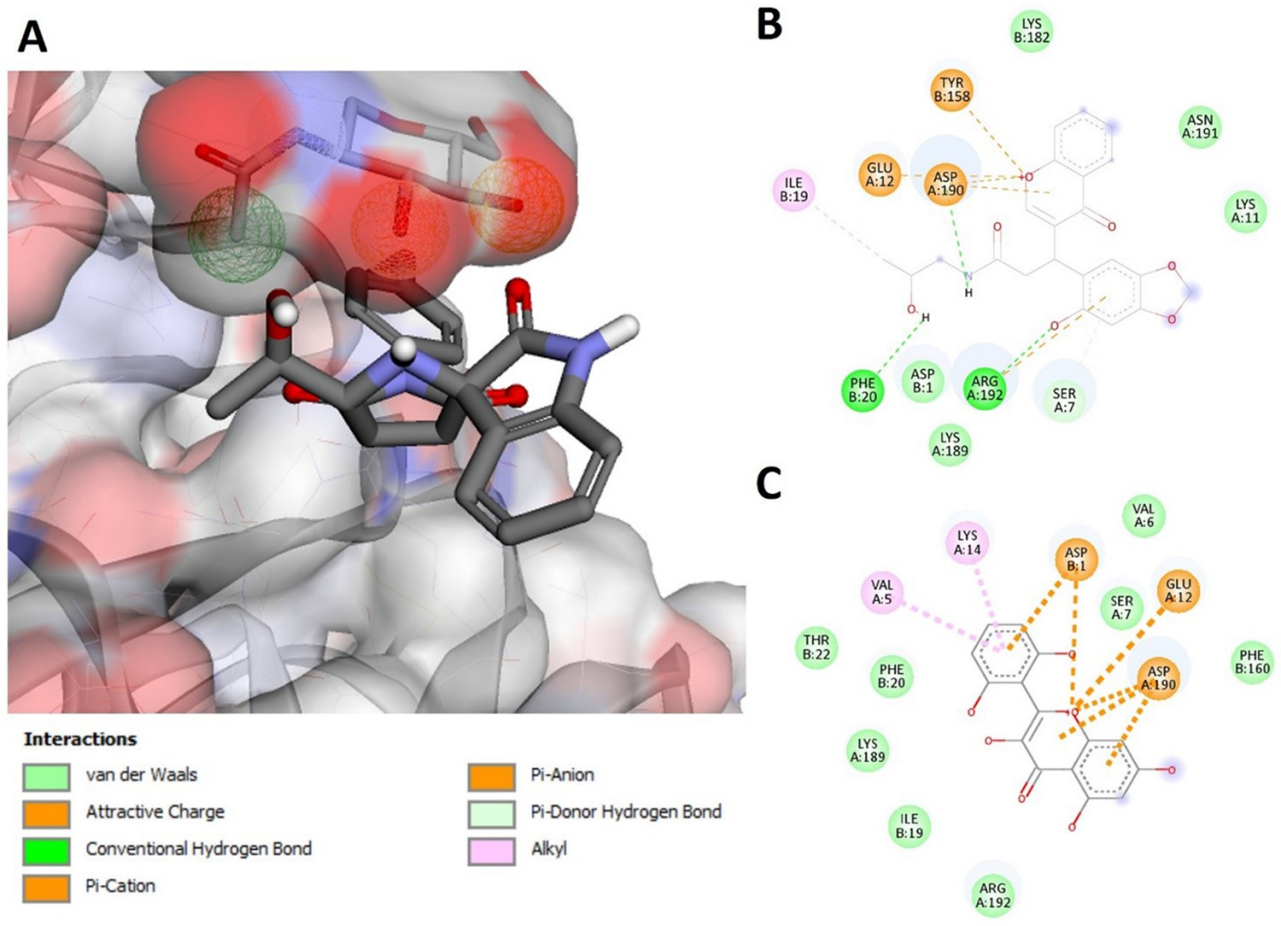
Top lead compounds docking in the active site of DENV-NS1. (A) Pharmacophore model designed for the DENV-NS1 and molecular interactions of top hit compounds according to ADME analysis with the binding pocket of NS1. (B) Molecular interactions of compound C2 with DENV-NS1. (C) Molecular interactions of compound C6 with DENV-NS1.

PPI network analysis revealed that DENV-NS1 had shown the greatest number of associations with human proteins in the network. The DENV-NS1 protein was subjected to pharmacophore-based virtual screening to identify suitable inhibitors among the millions of compounds available in the databases of Pharmit resource. The crystal structure of the DENV-NS1 protein (PDB ID: 4O6B) was used to obtain a pharmacophore model and for *in-silico* screening to discover lead inhibitors of NS1. After submission of the PDB code of NS1 to the Pharmit server, a set of interacting pharmacophore features were produced automatically from the ligand attached to the DENV-NS1 in the crystal structure. 22 pharmacophore features were identified by the Pharmit by default, and search query returned no result. A set of active and decoys were generated and submitted to Pharmit to identify the best features and validate the pharmacophore model. 5 essential features plus shape constraints were applied to build the model: 2 hydrogen donors, two hydrogen acceptors, and one hydrophobic. After repeated docking attempts, the top screened compounds were selected based on the lowest binding energy. The scores and RMSD values of the selected compounds are given in Table 3. The chemical structures and Molport IDs of the top hit compounds are shown in Fig 6. The obtained pharmacophore model based on the 3D structure of DENV-NS1 protein showed five pharmacophore features are shown in Fig 7. Fig 7A represents the pharmacophore model designed for NS1 using Pharmit server and was used to screen Molport. Top 8 hit compounds with lowest RMSD values were selected as mentioned in Table 4. Fig 7B and 7C shows the molecular interactions of the top hit compounds (MolPort-002-529-291 and MolPort-001-742-737) with the binding pocket of NS1.

**Table 3:** Top 8 best hit molecules based on docking scores and RMSD values.

S.No.	MolPort IDs	Molecular Weight	Pharmit Score	RMSD	Docking Score
1	MolPort-002-529-291	407.426	-4.26	2.888	-8.8
2	MolPort-044-180-163	411.41	-4.13	2.893	-8.1
3	MolPort-002-642-023	395.39	-3.94	2.998	-8.9
4	MolPort-001-838-033	381.428	-3.87	2.825	-8.2
5	MolPort-044-726-474	448.38	-3.44	2.961	-8.6
6	MolPort-001-742-737	302.238	-3.43	2.985	-8.0
7	MolPort-002-531-938	433.508	-3.36	2.904	-8.6
8	MolPort-000-650-550	393.439	-3.03	2.838	-8.3

**Table 4:** ADME analysis using SwissADME server.

Serial No.	Compounds (MolPort IDs)	H-Bond Acceptors	H-Bond Donors	TPSA (Å2)	Consensus Log P_o/w_	Molar Refractivity	GI Absorption	Log S	BBB Permeant	P-gp Substrate	Log Kp (skin permeation) cm/s	Drug likeness based on Lipinski rule	Bioavailability Score	PAINS (alert)	Brenk (alert)	Synthetic accessibility
C1	MolPort-002-529-291	6	3	107.97	0.86	117.67	High	-2.72 soluble	No	Yes	-8.50 cm/s	Yes; 0 violation	0.55	0 alert	1 alert: phthalimide	4.55
C2	MolPort-044-180-163	7	3	118.23	1.92	108.22	High	-3.41 soluble	No	Yes	-7.59 cm/s	Yes; 0 violation	0.55	0 alert	0 alert	4.34
C3	MolPort-002-642-023	6	3	98.74	1.18	111.13	High	-2.80 soluble	No	Yes	-8.34 cm/s	Yes; 0 violation	0.55	0 alert	1 alert: phthalimide	4.48
C4	MolPort-001-838-033	6	3	99.77	2.44	108.32	High	-3.23 soluble	No	Yes	-7.43 cm/s	Yes; 0 violation	0.55	1 alert: mannich_A	1 alert: cumarine	4.09
C5	MolPort-044-726-474	11	8	201.28	-0.24	108.63	Low	-2.70 soluble	No	No	-9.14 cm/s	No; 2 violations: NorO>10, NHorOH>5	0.17	1 alert: catechol_A	1 alert: catechol	5.04
C6	MolPort-001-742-737	7	5	131.36	1.21	78.03	High	-3.16 soluble	No	No	-7.05 cm/s	Yes; 0 violation	0.55	0 alert	0 alert	3.27
C7	MolPort-002-531-938	5	3	98.74	1.92	130.00	High	-3.50 soluble	No	Yes	-7.79 cm/s	Yes; 0 violation	0.55	0 alert	1 alert: phthalimide	4.97
C8	MolPort-000-650-550	6	3	99.77	2.40	110.39	High	-3.38 soluble	No	Yes	-7.48 cm/s	Yes; 0 violation	0.55	1 alert: mannich_A	1 alert: cumarine	4.05

### 3.5. Drug-likeliness, ADME assessment and molecular docking

**Fig 8:**
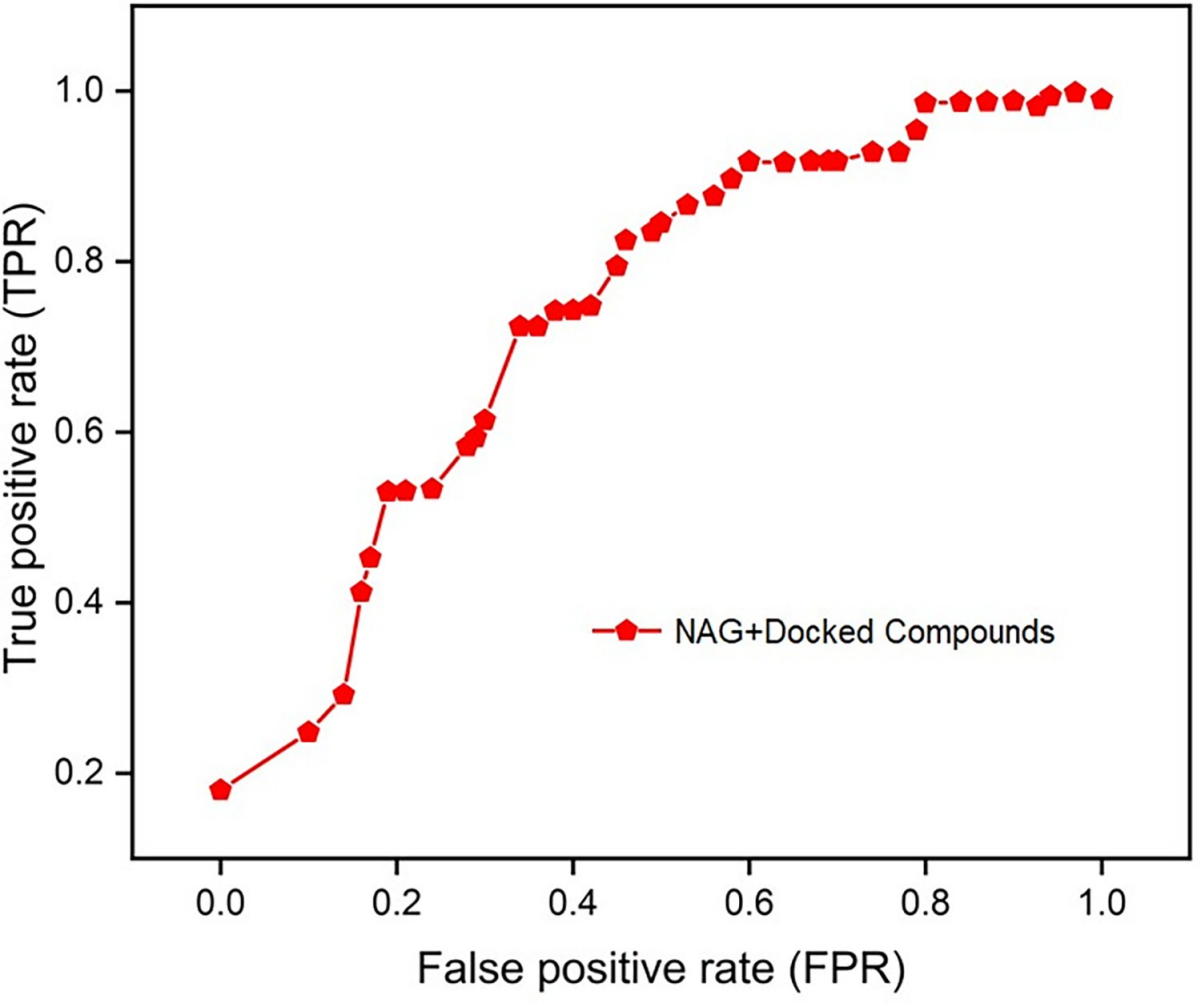
The ROC curve compares the success of the docking protocol of NAG ligand and the compound obtained during virtual screening. AUC of 0.68 was obtained based on binding affinity scores and interactions with critical residues. The ROC curve depicts the true positive rate (sensitivity) versus the false positive rate (1-specificity). OrigenLab was used to generate the graph.

To investigate the pharmacokinetic parameters of the retrieved molecular compounds, various parameters were computed, including logPo/w for octanol/water, molar refractivity (MR), permeability glycoprotein (P-gp), human oral absorption in the gastrointestinal tract (GI), aqueous solubility (log S), skin permeability coefficient (log Kp), drug-likeness, and the blood-brain barrier (BBB). Extremely hydrophobic compounds have minimal gut solubility and are solvated in the fat globules. As shown in Table 4, all compounds have drug-like features based on solubility levels. Except for MolPort-044-726-474, all compounds showed high absorption in the GI. According to the analysis, all compounds could not pass through the BBB. Lipinski’s rule of 5 is one of the most commonly used models for assessing therapeutic drug-like compounds based on their solubility and permeability [69]. To determine whether a compound has chemical and physical characteristics that would make it likely to be an orally active drug in humans, hit compounds were evaluated based on Lipinski’s rule of five. Among the top hit compounds, all compounds showed zero violation to Lipinski rule and have drug-like properties except for MolPort-044-726-474. The best fit compound according to ADME analysis was MolPort-044-180-163. However, these compounds can be chemically modified to exhibit additional drug-like properties. The compounds retrieved after virtual screening were docked into the active site of the DENV-NS1 protein and ranked according to their score. A greater negative AutoDock Vina score implies a significant binding affinity. The RMSD value was computed between NS1 and the re-docked inhibitors (Table 4 and Fig 7B, 7C). The docking results were validated using ROC statistics based on comparison between the binding confirmation of DENV-NS1 protein ligand (2-acetamido-2-deoxy-beta-D-glucopyranose) in the crystal structure and that obtained from docking studies with an AUC of 0.68 based on binding affinity scores and interactions with critical residues (Fig 8).

### 3.6. Molecular dynamic simulation

Based on ADMET analysis, we selected two compounds (C2; MolPort-044-180-163 and C6; MolPort-001-742-737) for further investigation. A 100-ns production run was performed to gain insight into the structural dynamics of the system. RMSD, RMSF, Rg, beta factor (β-factor), hydrogen bond occupancy (HBO), and protein-ligand contacts were plotted throughout the trajectory to assess conformational changes and stability of the structural components of the simulated complexes. Binding energies were also determined using the MMGBSA methods.

### 3.7. Stability analysis of DENV-NS1 protein

**Fig 9:**
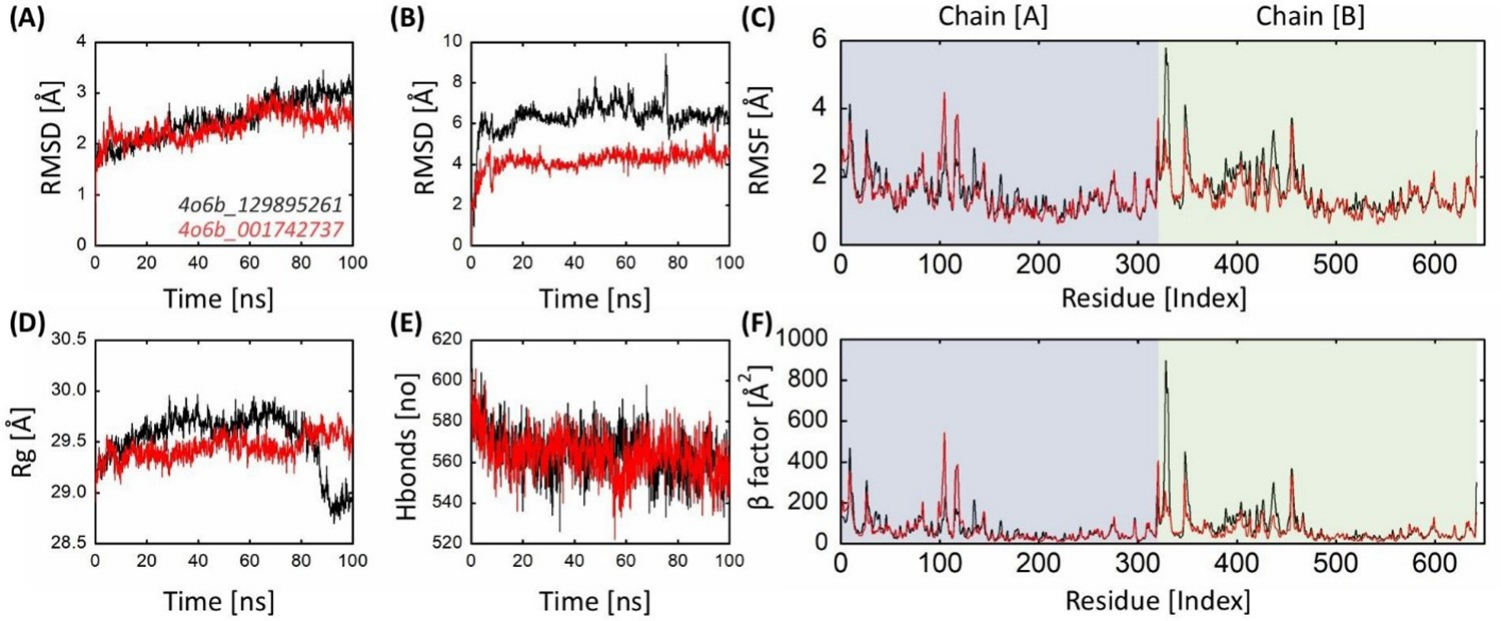
Post-simulation RMSD, RMSF, Rg, hydrogen bonds, and β-factor analysis of DENV-NS1 protein in complex with two drug-like compounds from the Molport database. (A) RMSD of the carbon-alpha of the bound-DENV-NS1 protein complex. (B) RMSD of the selected drug candidates from the Molport database. (C) RMSF of per-residue bound-DENV-NS1 protein. (D) Rg of bound-DENV-NS1 protein. (E) Inter-protein hydrogen bond occupancy of DENV-NS1 protein in complex with lead candidates. (F) Residue-based β-factor of DENV-NS1 protein in complex with lead candidates from the MolPort database.

For the C2 compound, when simulated, the average RMSD for the protein was recorded as 2.48 Å with a standard deviation of 0.43 and showed a stable plot, indicating no conformational changes occurred. The highest RMSD of the C2- DENV-NS1 complex was observed to be 3.45 Å. The average RMSD of C2 with respect to initial conformation of NS1 showed an average of 3.14 Å with a maximum of 4.4 Å which depicts that the ligand changed its position from the initial position. The Average RMSD of the DENV-NS1 in complex with C6 was 2.33 Å. The maximum RMSD was noted to be 3.07 Å (Fig 9A). The overall RMSD plot showed stability. This showed that the protein in the complex with C6 did not undergo any significant changes, whereas an abrupt change in the RMSD plots of C6 with respect to its initial position and C6 with respect to DENV-NS1 was observed. The analysis of the RMSD plot of the ligand with respect to initial conformation of NS1 for C6 showed the average RMSD of 2.09 Å with a maximum of 2.9 Å which depicts that the ligand changed its position from the initial position. This can also be clearly seen in the RMSD plot of the ligand with respect to the protein (Fig 9B).

### 3.8. Flexibility analysis of DENV-NS1 protein upon binding

The compound C2 and C6 in complex with DENV-NS1 exhibited the mean RMSF of 1.44 Å and 1.56 Å respectively (Fig 9C). The residue Thr318 showed the highest flexibility of 6.31 Å for C2, while Arg105 had the highest RMSF of 4.99 Å for C6. Likewise, Pro219 had the lowest RMSF values of 0.59 Å for C2, while Asp542 showed the lowest RMSF value of 0.56 Å for C6. The RMSF values of both complexes were very similar. The terminal residues were highly flexible and exhibited the highest fluctuation. The most discernible difference between the RMSF plots of all complexes was in Thr318 and Arg105. In addition, the sets of Gly116, Leu117, and Glu118 residues showed notable peaks in the RMSF plots. These residues (Gly116, Leu117, and Glu118) are residues of the binding pocket, which is comprised of loops. This fluctuation may be due to the binding of the compounds. The superimposed RMSF plots of all complexes are shown in Fig 9C.

### 3.9. Compactness of DENV-NS1 protein via gyration analysis

The compactness of the system was investigated using the radius of gyration. A lower value of the radius of gyration indicates greater compactness. The average values for the radius of gyration for all complexes were very close and showed only minor differences, indicating that the compacts of all the systems were similar. The average values of radius of gyration for compound C2 and C6 bound to DENV-NS1 were 29.52.76 Å, and 29.44 Å, respectively (Fig 9D). In comparison, the C6 complex showed convergence, whereas C2 showed non-uniform compactness.

### 3.10. Inter-protein hydrogen bond occupancy of DENV-NS1 protein

Hydrogen bonds are crucial mediators with high affinity and specificity for ligand binding to proteins. This study also analyzed the hydrogen bond profile/occupancy of the complexes under study throughout the simulation (Fig 9E). As shown in Fig 9E, the complexes exhibit hydrogen bonding, ranging from 522 to 606. C2 displayed 526–606 hydrogen bonds, while C6 displayed 522–606 hydrogen bonds during the simulation trajectory. The average number of hydrogen bonds was 564.6 for C2 while 563.6 for C6. It can be concluded that C2 (when bound to DENV-NS1) showed maximum hydrogen bonding, while C6 showed minimum hydrogen bonding; however, the difference was minimal.

### 3.11. β-factor anomalies of DENV-NS1 over the trajectory

The B-factor, also known as thermal disorderedness, calibrates a function that assesses structural stability in terms of RMSF at the atomic site. The β-factor and the RMSF are two parameters that complement each other. The DENV-NS1 in complex with C2 and C6 showed the average β-factor of 76.89 Å^2^ and 64.69 Å^2^, respectively (Fig 9F). The highest average β-factor was revealed by C2, while the C6 showed the lowest average β-factor value and was the most stable among all the complexes.

### 3.12. Changes in secondary structure elements (SSE) of DENV-NS1 receptor

**Fig 10:**
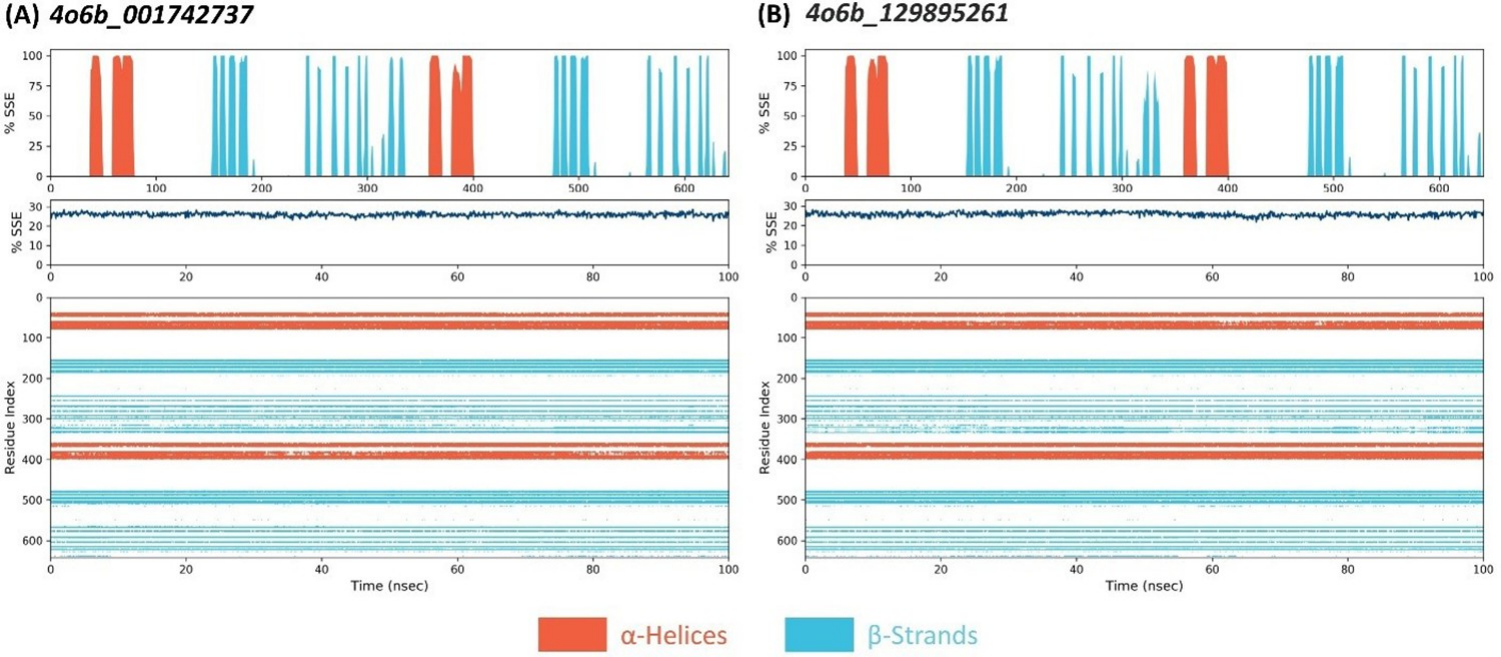
Residue wise percentage SSE analysis of DENV-NS1 protein and SSE contribution of each residue as function of time for 100ns simulation trajectory (A) Per-residue percentage of the DENV-NS1 protein when bound to Molprot_001742737. (B) Per-residue percentage of the DENV-NS1 protein when bound to Molprot_129895261.

Protein secondary structural elements (SSE) comprising α-helices and β-strands were analyzed throughout the simulation. Fig 10 depicts the distribution of SSE by complexes over the protein structure. The structural region of the protein is firmer, as shown in the graph. The SSE of all complexes exhibited minor differences. The major structural component of DENV-NS1 is composed of α-helices. The total SSE% for DENV-NS1 when bound to C2 and C6 was 25.84% and 26.08%, respectively. Typically, the terminal regions (N-terminal and C-terminal) of proteins fluctuate more than any other portion. In contrasted to the loop regions, α-helices and β-strands (secondary structural elements) deviated less (Fig 10). The unstructured region of the protein exhibited a lower stiffness.

### 3.13. DENV-NS1 protein contacts with novel drug candidates

**Fig 11:**
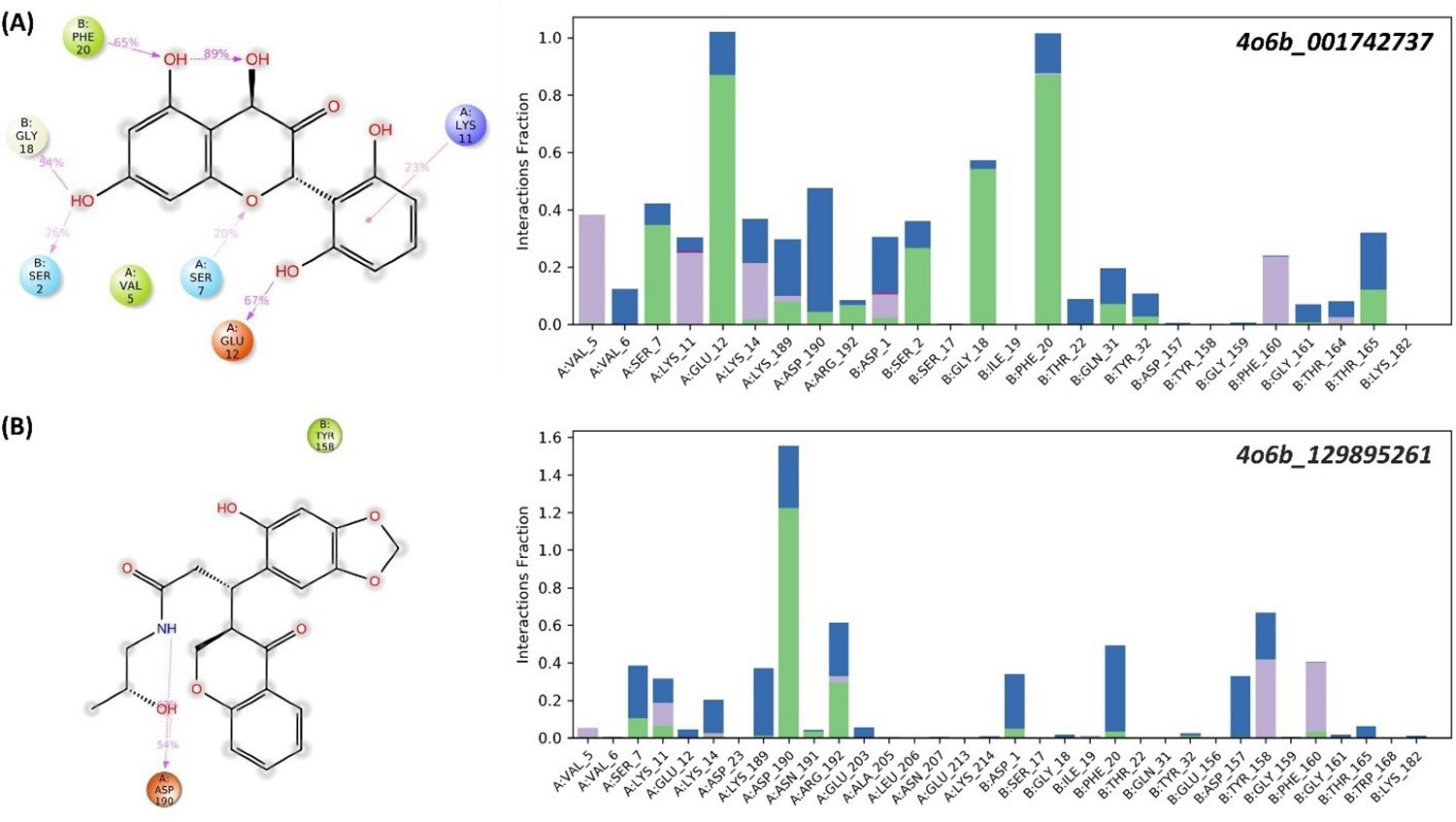
Protein-ligand contacts of DENV-NS1 protein in complex with two drug-like candidate compounds from the Molprot database. (A) Residue wise protein-ligand contacts of the bound- DENV-NS1 protein with Molport_001742737. (B) Residue-wise protein-ligand contacts of the bound-DENV-NS1 protein with Molport_129895261.

The protein-ligand interactions of the receptor-ligand complex were examined using protein-ligand contact histograms. Hydrogen bonds, hydrophobic interactions, ionic interactions, and water bridge interactions were used to classify the protein-ligand contacts. Hydrogen bonding plays an important role in the binding of ligands to proteins. The importance of hydrogen bonding properties in drug design emerges from their considerable influence on drug selectivity, metabolism, and adsorption. A wide range of residues were involved in hydrogen bonds and hydrophobic interactions with the C6. Residues Ser7, Lys11, Glu12, Gly18, and Phe20 created hydrogen bonds with C6 (Fig 11A). In addition to this, residues Glu12 and Phe20 formed more promising interactions throughout the simulation. Val5 and Phe20 were involved in hydrophobic contacts with C6, and many of these residues also formed water bridges with the ligand. Compound C2 formed hydrogen bonds with the residue Asp90. Hydrophobic residues, such as Tyrosine (Tyr) residues, were observed to be highly involved in hydrophobic contacts with C2 (Fig 11B). Ser7, Lys11, and Glu12 are conserved interactions between both complexes.

### 3.14. Binding energy of DENV-NS1 against novel drug candidates

**Fig 12:**
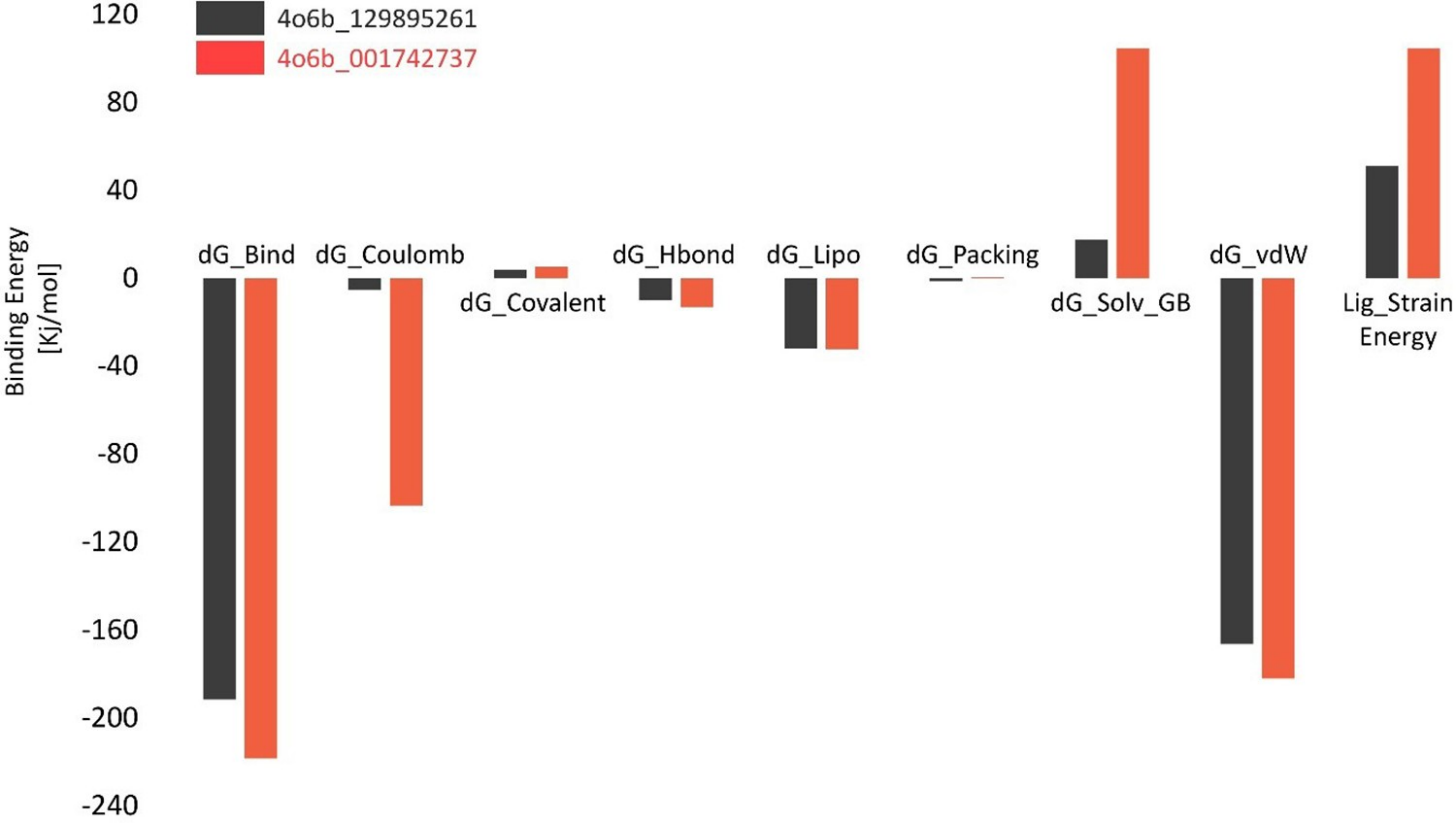
Binding energy decomposition analysis of bound- DENV-NS1 protein with novel drug candidates using the MMGBSA approach.

Receptor and ligand-binding free energies are excellent predictors of their interactions. The MMGBSA approach successfully simulates the capacity of a ligand to bind to a protein. The binding energy was computed for all complexes utilizing the prime MMGBSA tool. The energies of all complexes are listed in Table 1. All compounds showed better results and maximum binding energies than the control. The G-binding energy values of the selected compounds (C2 and C6) varied between -191.68 and -218.39 kj/mol. The total binding energies for C2 and C6 were -191.68 kJ/mol and -218.39 kJ/mol, respectively. The C6 exhibited least G-bind energy as compared to C2 complexes. To further investigate about the energy components that lead to more efficient binding within protein-ligand complexes, the electrostatic, H-bond, van der Waals, and ligand strain energies were calculated. The total binding energy, van der Waals force, polar solvation energy, Coulomb force, and covalent force were all favorable for C6, which can also be seen in the bar graph (Fig 12). All DENV-NS1 complexes exhibited advantageous binding due to the van der Waals energy; however, the protein-ligand interaction was significantly inhibited by the Solv GB (Generalized Born electrostatic solvation energy). For these compounds, the Solv GB range was 17.62 to 104.53 kj/mol whereas the Van der Waals energy varied from -166.36 to -112.07 kj/mol. Both were rather large, but they counteract each other to create overall positive total electrostatic contributions.

## 4.0. Discussion

The results explained in the previous sections of this study reveal that the top 5 and 30 highly dengue-associated gene products are enriched in various other pathways, indicating that these proteins are not well studied in dengue viral infections. The top 5 proteins with the highest number of interactions with dengue proteins in the network were associated with another insect-borne disease, African trypanosmiasis. Other disease pathways included malaria, mitophagy, and prostate cancer. Enrichment analysis of the gene sets reveals that the Dengue fever is not the only disease in which the genes are actively involved. HBA1, UBE2I, CSNK2A1, RRP12, and HSP90AB1 were highly enriched in African trypanosmiasis (sleeping sickness) than in the dengue viral infection pathway. Gene Ontology analysis of the top 30 highly associated human proteins identified significant biological processes in which the set of proteins actively played a part. Biological regulation, metabolism, cellular component organization, response of cellular activities to different stimuli, cellular localization, and developmental processes are the main biological processes involving a set of genes.

In the current approach, we present 1195 unique interactions between dengue-human proteins by integrating all previous small-scale and large-scale studies done on PPI of Dengue virus. In our study, we included every single interaction detected in the experimental methods used to identify the PPIs. For instance, Srisutthisamphan *et al*. (2018) detected the interaction between human heat shock protein 90 (Hsp90) and 6 DENV proteins using co-immunoprecipitation experiments. The manual curation of research studies is the most trusted approach for obtaining data. Several PPI databases curate data from the published literature and keep up with new research in this field. A network-based study of the infection of host with viral pathogenesis has also progressed over time. Recently, Zheng *et al*. (2021) [70] have performed various analysis to study the pathological mechanisms of the coronavirus disease 2019 and dengue virus co-infection using network-based approaches and identified 460 common core targets between them. Viral genomes code for a very small number of proteins compared to other pathogens, makes it easy to understand the mechanism of infection with viruses. In our previous studies, we constructed and analyzed comprehensive protein interaction networks of Hepatitis C Virus, Influenza A virus, and human papillomavirus with their host Homo sapiens [18] and found many crucial insights that helped us to find potential targets against infections caused by HCV, IAV, HPV, and some other disease pathways.

In this study, we prioritized DENV-NS1 as a potential therapeutic drug target based on the highest number of PPIs with human host. DENV-NS1 has been reported to cause endothelial hyperpermeability in human dermal, lung, and umbilical cells in vitro and vascular extravasation in lung, liver, and small intestine in mouse models [71, 72]. Recently, Menezes et al. (2022) targeted the NS1 protein of three different Flavivirus species including DENV, Zika virus (ZIKV), and Yellow fever virus (YFV) [73]. They performed virtual screening of small natural compounds from the ZINC database followed by molecular docking and molecular dynamic simulations to identify a common drug to target the NS1 protein of all three viruses. We performed pharmacophore-based virtual screening of millions of small drug-like compounds available in multiple public database resources to identify the best possible inhibitor of DENV-NS1. The top 10 lead compounds were selected after scanning millions of compounds in the available databases. The compounds were further narrowed down to 8 based on energy minimization and Pharmit scores. The redocking of these top-hit compounds was further validated by ROC statistics, which suggested the successful binding of these compounds to the binding pocket of DENV-NS1. Based on physicochemical and pharmacokinetic properties, compounds C2 (MolPort-044-180-163) and C6 (MolPort-001-742-737) were determined as the top ranked molecules. These compounds showed promising drug-like capabilities; in particular, both compounds followed Lipinski’s Rule and may be considered as potent inhibitors of DENV-NS1. The intermolecular interactions of the DENV-NS1-C2/C6 complexes exhibited significant interactions involving conserved residues, suggesting the importance of these residues in the high binding affinity of these compounds and their biomolecular properties. These drug-like compounds bind to the same binding site of the DENV-NS1 protein; therefore, their entropic contribution would also be similar. The constantly lower binding free energy during the MD trajectory analysis of the DENV-NS1-C2/C6 complexes also displayed structural stability, implying that these molecules will show stable interactions in a cellular environment. As a result, this method can be promiscuous in prediction and comparison of relative binding energies in biomolecular complexes. These *in-silico* validated results need to be investigated using experimental and clinical assays before classifying these molecules as putative DENV inhibitors. The *in-silico* approaches used in this study could pave the path for the identification of novel therapeutic drugs against different pathogens and could lead to the development of specie-specific potential drugs that could aid in the elimination of deadly viral infections.

## 5.0. Conclusions

In this study, we adopted state-of-the-art system biology techniques with molecular docking and molecular dynamic simulations to identify druggable protein candidates for DENV. A protein interaction map of the dengue virus and associated host proteins was constructed from experimental data. KEGG pathway and gene ontology analyses of the highly Dengue virus-associated human proteins identified proteins involved in biological processes targeted by the virus. The DENV-NS1 protein was prioritized as a druggable protein based on the highest number of interactions with the human host. Pharmacophore-based virtual screening of small natural compounds was performed to identify potential DENV inhibitors. Molecular docking and MD simulation studies were carried out to validate the strong binding affinities, flexibility, and stability of the DENV-NS1-C2/C6 complexes in the cellular environment. Experimental and clinical assays are required to validate the results of this study further.

## Supporting information

S1 Table High scoring interacting proteins of dengue virus. (XLSX)

S2 Table List of human proteins with greatest number of associations with DENV proteins. (DOCX)

S1 Fig KEGG pathway enrichment analysis of the top 30 highly DENV-associated human proteins. The bar chart shows that the proteins are highly enriched in protein export pathway (enrichment ratio: 0.04). Other pathways include prion diseases, thyroid hormone synthesis, african trypanosmiasis, NF-kappa B signaling pathway and several other disease pathways including measles, malaria and primary immunodeficiency. Excelsheet S1 containing all the interacting proteins of human and DENV proteins.
(PNG)

S1 Graphical abstract (JPG)

## Abbreviations

DENV: Dengue Virus
C: Capsid protein
E: Envelope protein
M: Membrane protein
NS1: Non-structural protein 1
NS2A: Non-structural protein 2A
NS2B: Non-structural protein 2B
NS3: Non-structural protein 3
NS4A: Non-structural protein 4A
NS4B: Non-structural protein 4B
NS5: Non-structural protein 5
HBA1: Hemoglobin alpha locus 1
UBE2I: Ubiquitin Conjugating Enzyme E2 I
PPI: Protein-Protein Interaction
KEGG: Kyoto Encyclopedia of Genes and Genomics
