# Supplementary material for: A comprehensive protein interaction map and druggability investigation prioritized dengue virus NS1 protein as promising therapeutic candidate
Source: PLoS One. 2023 Jul 27;18(7):e0287905. doi: 10.1371/journal.pone.0287905 (PMC10374080; doi:10.1371/journal.pone.0287905)
Supplement: S2 Table — (DOCX) [file pone.0287905.s002.docx]

Table S1: High scoring interacting proteins of Dengue virus.

| DENV proteins | No. of interactions | Percentage (from total no. of interactions) |
| --- | --- | --- |
| NS1 | 430 | 36% |
| NS5 | 229 | 19% |
| NS3 | 202 | 17% |

Table S2: List of human proteins with greatest number of associations with DENV proteins.

| Human proteins | No. of interactions with DENV proteins | Associated DENV proteins |
| --- | --- | --- |
| HBA1  UBE2I | 7 | E, Capsid, NS5, NS2B, NS3, NS4B, NS4A  E, Capsid, NS2A, NS5, NS2B, NS3, NS4B |
| CSNK2A1  RRP12  HSP90AB1 | 6 | E, Capsid, NS2A, NS3, NS4B, NS1  M, E, Capsid, NS5, NS3, NS1  E, NS5, NS2B, NS3, NS4B, NS1 |
| IKBKE  GAB1  ZNF410, LRRFIP1, FAM192A,HSPA5  PSMC1  BOD1L  ANP32B, NAP1L1, AP3B1, OS9 | 5 | NS2A, NS2B, NS3, NS4B, NS4A  E, Capsid, NS4B, NS4A, NS1  M, E, Capsid, NS5,NS3  E, Capsid, NS5, NS3, NS1  Capsid, M, NS1, NS3, NS5  Capsid, E, NS1, NS3, NS5 |


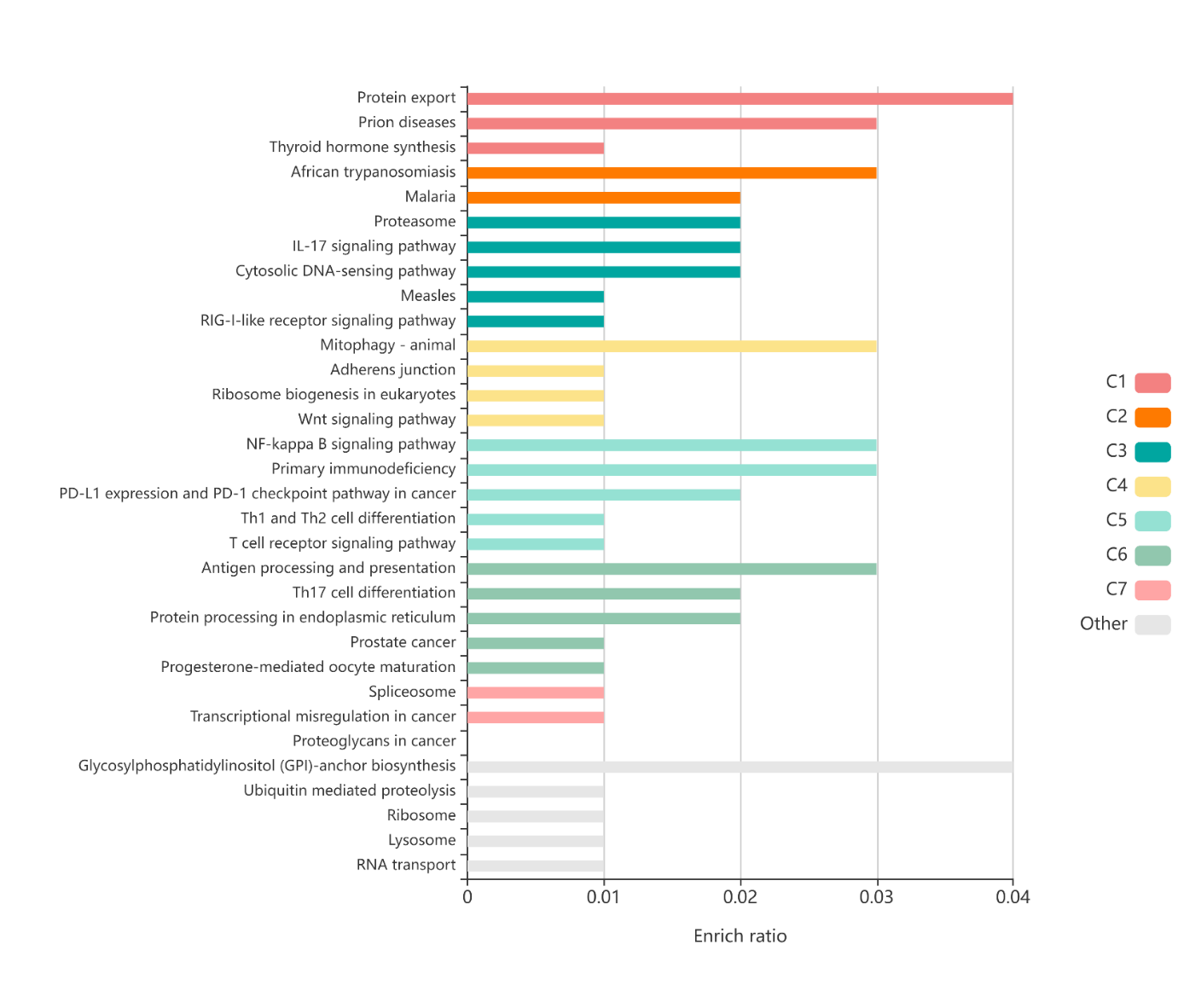


Figure S1: KEGG pathway enrichment analysis of the top 30 highly DENV-associated human proteins. The bar chart shows that the proteins are highly enriched in protein export pathway (enrichment ratio: 0.04). Other pathways include prion diseases, thyroid hormone synthesis, african trypanosmiasis, NF-kappa B signaling pathway and several other disease pathways including measles, malaria and primary immunodeficiency.
