# Supplementary figures and images for: A comprehensive protein interaction map and druggability investigation prioritized dengue virus NS1 protein as promising therapeutic candidate
Source: PLoS One. 2023 Jul 27;18(7):e0287905. doi: 10.1371/journal.pone.0287905 (PMC10374080; doi:10.1371/journal.pone.0287905)

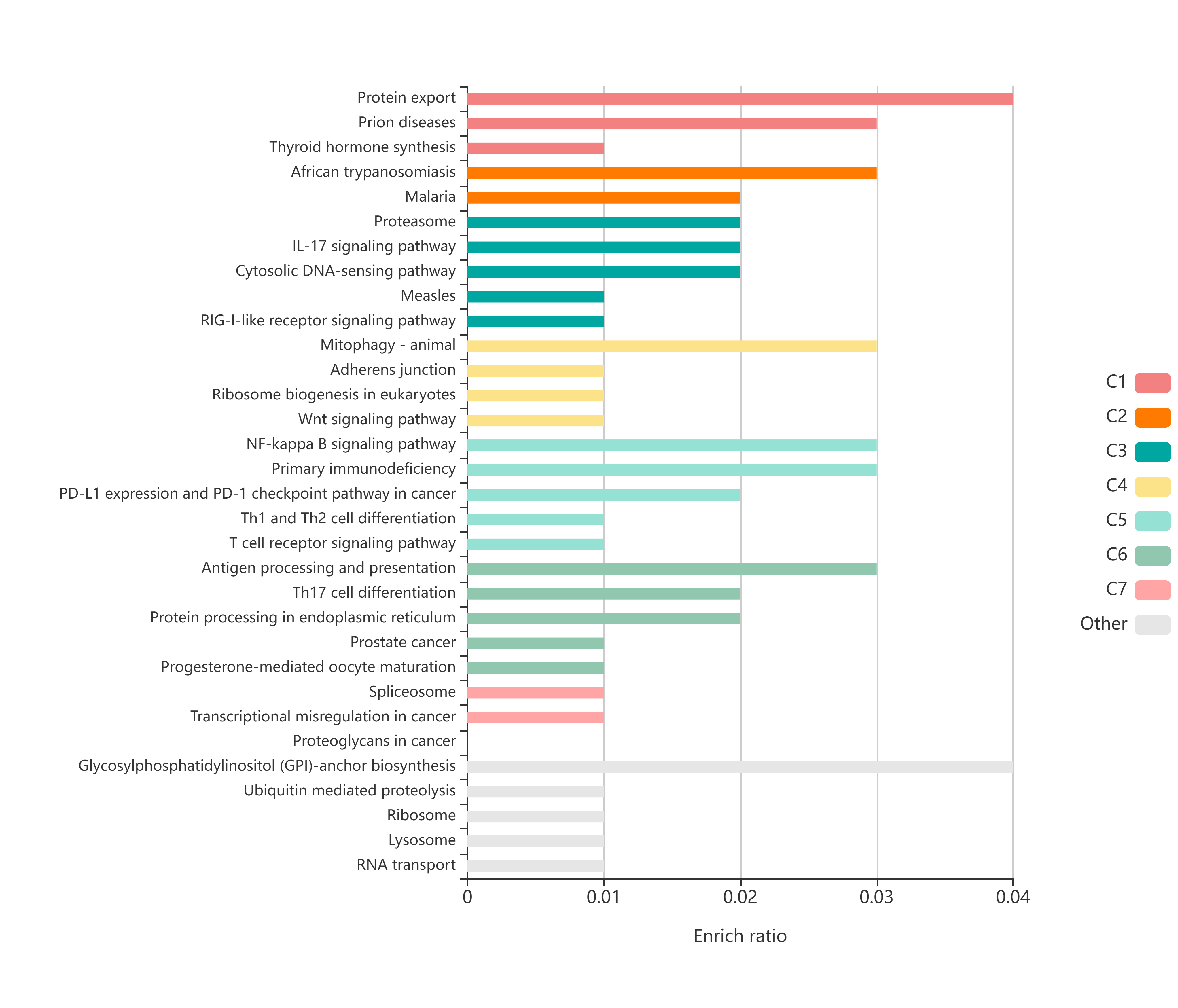

Supplement: S1 Fig — The bar chart shows that the proteins are highly enriched in protein export pathway (enrichment ratio: 0.04). Other pathways include prion diseases, thyroid hormone synthesis, african trypanosmiasis, NF-kappa B signaling pathway and several other disease pathways including measles, malaria and primary immunodeficiency. Excelsheet S1 containing all the interacting proteins of human and DENV proteins. (PNG) [file pone.0287905.s003.png]

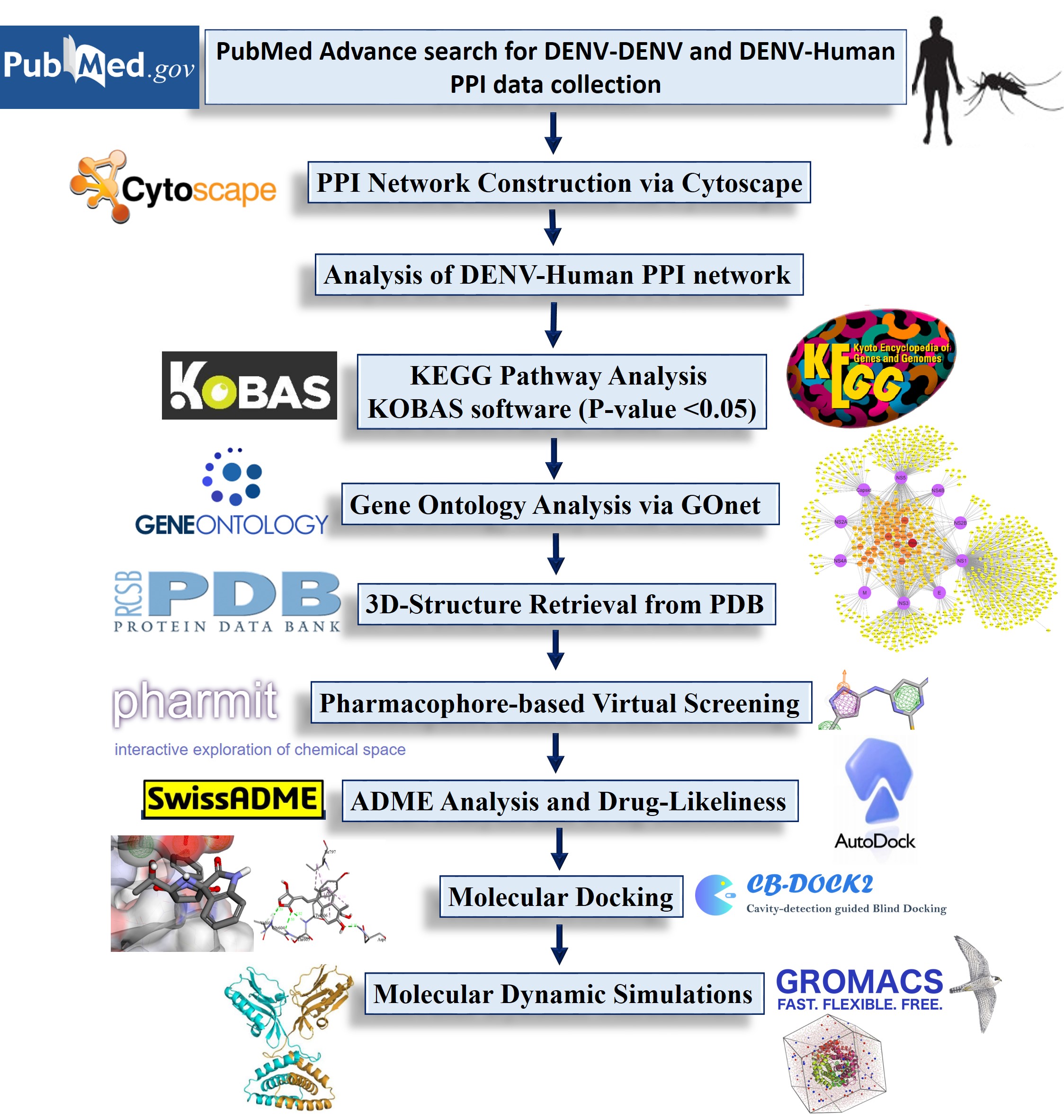

Supplement: S1 Graphical abstract — (JPG) [file pone.0287905.s004.jpg]
